# Corrosion Rate and Mechanism of Degradation of Chitosan/TiO_2_ Coatings Deposited on MgZnCa Alloy in Hank’s Solution

**DOI:** 10.3390/ijms25105313

**Published:** 2024-05-13

**Authors:** Halina Krawiec, Iryna Kozina, Maria Starowicz, Maria Lekka, Caterina Zanella, Lorenzo Fedrizzi, Michele Fedel, Flavio Deflorian

**Affiliations:** 1 Faculty of Foundry Engineering, AGH University of Krakow, 23 Reymonta Street, 30-059 Krakow, Poland; 2 Polytechnic Department of Engineering and Architecture, University of Udine, 33100 Udine, Italy; mlekka@cidetec.es (M.L.);; 3 Department of Materials and Manufacturing, Jonkoping University, 553 18 Jonkoping, Sweden; 4 Department of Industrial Engineering, University of Trento, 38123 Trento, Italy

**Keywords:** magnesium alloy, chitosan, coating, TiO_2_ nanoparticles, corrosion rate

## Abstract

Overly fast corrosion degradation of biodegradable magnesium alloys has been a major problem over the last several years. The development of protective coatings by using biocompatible, biodegradable, and non-toxic material such as chitosan ensures a reduction in the rate of corrosion of Mg alloys in simulated body fluids. In this study, chitosan/TiO_2_ nanocomposite coating was used for the first time to hinder the corrosion rate of Mg19Zn1Ca alloy in Hank’s solution. The main goal of this research is to investigate and explain the corrosion degradation mechanism of Mg19Zn1Ca alloy coated by nanocomposite chitosan-based coating. The chemical composition, structural analyses, and corrosion tests were used to evaluate the protective properties of the chitosan/TiO_2_ coating deposited on the Mg19Zn1Ca substrate. The chitosan/TiO_2_ coating slows down the corrosion rate of the magnesium alloy by more than threefold (3.6 times). The interaction of TiO_2_ (NPs) with the hydroxy and amine groups present in the chitosan molecule cause their uniform distribution in the chitosan matrix. The chitosan/TiO_2_ coating limits the contact of the substrate with Hank’s solution.

## 1. Introduction

Magnesium and its alloys have received a lot of attention in recent decades. Magnesium alloys are ultralight, exhibit good strength characteristics, and have excellent vibration damping properties. These properties ensure their use in many application sectors (automotive, aerospace, energy, military, medicine, etc.) [1,2,3,4]. Magnesium and its alloys can also be used as temporary bone implants. Screws and plates made of magnesium alloys degrade over time in the body fluid environment and, after a certain time, disappear. Thus, surgical re-operation is not necessary to remove the implant [3].

The very high corrosion rate of magnesium and its alloys is a huge limitation in their application. Due to the very low electrochemical potential, magnesium and its alloys are susceptible to corrosion in aqueous electrolytes, particularly in chloride solutions and human body fluids. Corrosion of Mg alloys immersed in physiological fluids causes hydrogen evolution and environment alkalisation [5,6].

The corrosion resistance of magnesium can be improved by alloying additives such as Zn, Ca, Li, Mn, and rare earth elements [2,7,8,9]. Moreover, surface treatments and coatings are used to delay the degradation of magnesium alloys. Surface modification can be carried out in different ways, such as micro-arc oxidation [10,11,12] and conversion coating [13,14]. In recent years, the application of chitosan in the corrosion protection of magnesium alloys has been widely studied [6,14,15,16,17,18,19,20]. As a natural, non-toxic, biocompatible, biodegradable polymer compound, chitosan is widely used in medicine, food, cosmetics, and many other fields. Chitosan can also be modified with other compounds or nanoparticles to increase its functionality [15,16,17,18,19,20].

The biocompatibility and the corrosion resistance of AZ31 magnesium alloy was improved by aerosol deposition of hydroxyapatite (HA)–chitosan composite coatings [20]. Some researchers [17,19] revealed that the presence of a multi-layered coating significantly slows down the corrosion rate in simulated body fluid; this result was found for many magnesium alloys. Bi-layered functional coatings, consisting of an internal silane-TiO_2_ and external chitosan or collagen coatings, were designed to slow down the corrosion rate of AZ31 and ZE41 Mg alloys [17]. An inner silane-TiO_2_ coating was designed to slow down the corrosion rate, while the top layer composed of collagen or chitosan improves the biocompatibility and bioactivity. It was revealed that when collagen or chitosan top layers are present, corrosion products such as MgCO_3_ and CaCO_3_ are formed, providing additional corrosion protection to the Mg alloys in the simulated body fluids. A significant decrease in the corrosion rate of AZ31B magnesium alloy was also achieved by application of chitosan/heparinised graphene oxide (Chi/HGO) multilayer coatings on its surface [19]. Heparin is a substance with excellent biocompatibility, and it was used for the graphene oxide modification. The (Chi/HGO) multilayer coatings simultaneously enhance the corrosion resistance and improve the biocompatibility of magnesium alloys. The corrosion rate of Mg20Zn alloy was reduced by a factor of 2.6 by deposition of two coatings on its surface. The internal calcium phosphate coating was covered with a chitosan coating modified with the addition of water glass. A Mg20Zn alloy coated with a double coating (Ca-P/chitosan with water glass) showed very good corrosion resistance, which was due to the formation of CaSiO_3_ and Ca_3_(PO_4_)_2_ compounds on its surface. These compounds exhibit very low solubility in water; therefore, they effectively block the surface of the magnesium alloy against corrosion in the Hank’s solution [6].

The composite coatings based on chitosan and TiO_2_ show a wide range of applications. Titanium dioxide nanoparticles exhibit antimicrobial properties—they are used for photo-catalysis and water treatment [21,22]. It was shown that chitosan-TiO_2_ composite coatings can enhance the corrosion resistance of aluminium and stainless steel [23,24]. The very good corrosion resistance of CS-TiO_2_-coated aluminium was due to the formation of a cross-linked Al-O-Ti-O-Si-O-Si structure on its surface. The corrosion tests for uncoated and coated aluminium were performed in 3.5% NaCl solution. In the case of X2CrNiMo17-12-2 stainless steel, the increase in the corrosion resistance was due to a significant reduction in the exposure area between the stainless steel and Ringer’s solution.

The aim of this work was to investigate the corrosion rate and propose a corrosion mechanism for uncoated and chitosan/TiO_2_-coated magnesium alloy Mg19Zn1Ca. Furthermore, the degradation time of the chitosan/TiO_2_ coating in Hank’s solution was determined, and the role of TiO_2_ nanoparticles in slowing down the corrosion rate of the magnesium alloy was clarified. All the alloying elements present in Mg19Zn1Ca alloy are non-toxic, biocompatible, and biodegradable [8,25,26]. Calcium improves the mechanical strength and refines grains, whereas zinc elevates the corrosion potential of magnesium alloy in the simulated body fluid and reduces its degradation rate. Furthermore, the structure of corrosion products was investigated after corrosion tests.

## 2. Results and Discussion

### 2.1. Structure of the Substrate and the Chitosan/TiO_2_ Coating

**Figure 1:**
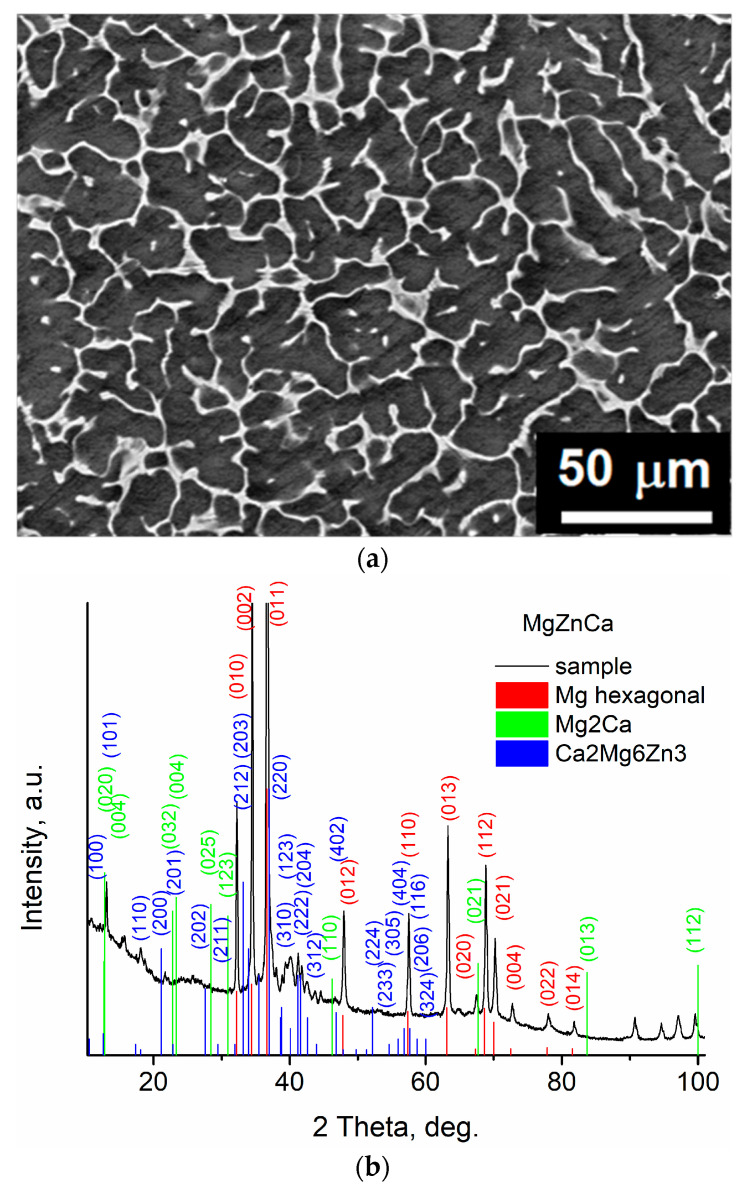
(**a**) Microstructure of Mg19Zn1Ca alloy revealed after mechanical polishing, (**b**) XRD diffraction pattern of Mg19Zn1Ca.

Figure 1a shows a FE-SEM top-view image of the microstructure of Mg19Zn1Ca alloy. An intermetallic phase is visible along the grain boundaries. EDS analysis reveals that the matrix is composed of magnesium (98.0 ± 0.7 at.%) with a small amount of zinc (2.0 ± 0.2 at.%). The intermetallic phase is composed of magnesium (61.0 ± 3.0 at.%), zinc (35.0 ± 4.0 at.%) and calcium (3.8 ± 1.0 at.%). XRD measurements show the presence of three phases, namely a α-Mg hexagonal close-packed crystal structure (corresponding to the matrix), Mg_2_Ca and Ca_2_Mg_6_Zn_3_ (both corresponding to intermetallic phases), Figure 1b. The peak observed at a 2θ angle of 36.5° is related with the α-Mg hexagonal phase [27,28].

**Figure 2:**
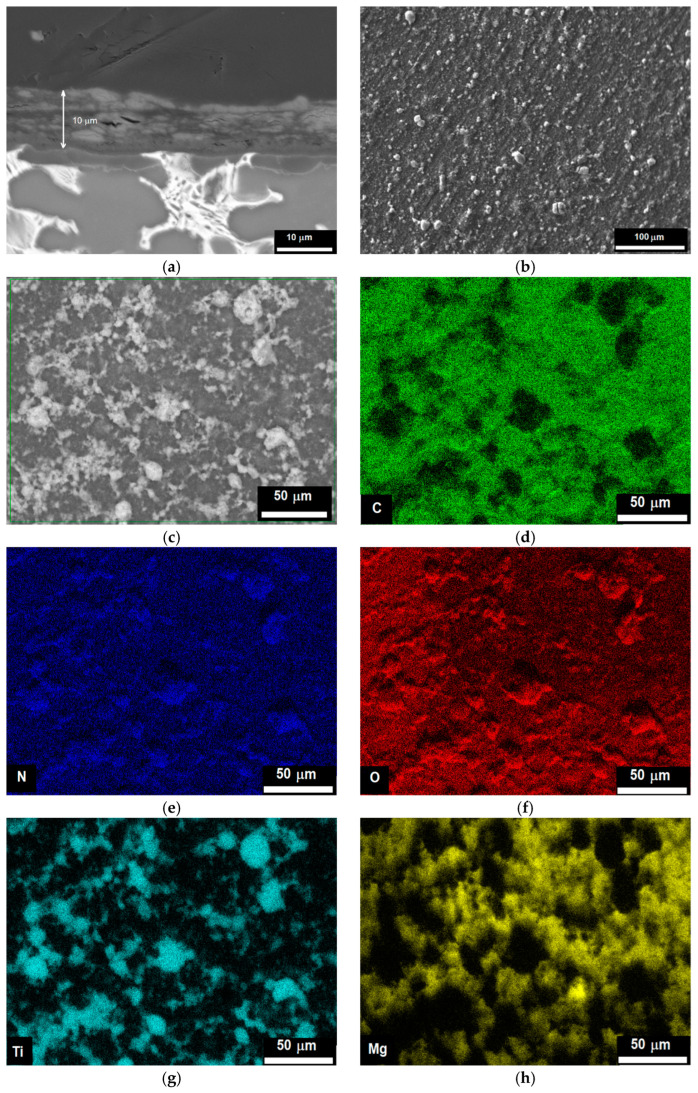
SEM images of TiO_2_ coating deposited on Mg19Zn1Ca substrate: (**a**) cross section, (**b**,**c**) top view, and (**d**–**h**) mapping of the elements present in the coating.

The morphology of the chitosan/TiO_2_ coating was investigated using SEM. Figure 2a shows a FE-SEM cross-section image of the sample covered with the chitosan/TiO_2_ coating. The coating is uniformly deposited on the substrate without defects at the interface. Its thickness is about 10 µm. The surface roughness (R_a_) of the coating measured by means of optical profilometry is 900 nm. The FE-SEM top view image of the chitosan/TiO_2_ coating is shown in Figure 2b,c. The TiO_2_ nanoparticles appear in the white colour and are uniformly distributed in the chitosan matrix.

The distribution of carbon, nitrogen, oxygen, titanium, and magnesium on the coating surface is presented in Figure 2d–h. It can be noticed that titanium and nitrogen are mainly present in sites where TiO_2_ nanoparticles are located, Figure 2e,g. By contrast, oxygen is uniformly distributed in the coating, Figure 2f. Carbon and magnesium are located around TiO_2_ nanoparticles, Figure 2d,h. The presence of a higher amount of nitrogen in sites containing TiO_2_ nanoparticles suggests that there is an interaction between amino groups (NH_2_) from chitosan molecules and titanium dioxide. Titanium, whose atomic orbital d is not completely full, can be an acceptor of a lone pair of electrons from an atom of another element. Nitrogen atoms have a lone pair of electrons and can give it to titanium. It was revealed [29] that the formation of TiO_2__N_x_ nanoparticles can be obtained at room temperature by direct amination of titanium dioxide (TiO_2_) nanoparticles.

**Figure 3:**
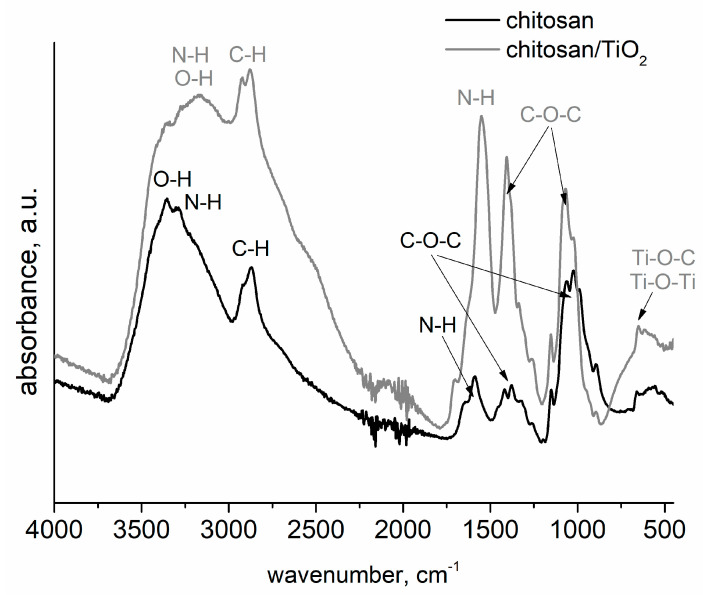
FTIR spectra of the chitosan and chitosan/TiO_2_ coatings.

The molecular structure of chitosan and chitosan/TiO_2_ coating was investigated by means of FT-IR spectroscopy. Figure 3 shows the FT-IR spectra obtained for chitosan and chitosan/TiO_2_ coatings. In the chitosan spectrum (black curve, Figure 3), the main bands appearing in the range from 3358 cm^−1^ to 3285 cm^−1^ are attributed to stretching vibration of hydroxyl (OH) and N-H groups. The bands visible at 2930 cm^−1^ and 2877 cm^−1^ are attributed to C-H bond in (-CH_2_), and -CH_3_ groups, respectively [30,31]. The band at 1590 cm^−1^ is related to -NH group with bending vibration, [32]. Moreover, the bands appearing at 1386 cm^−1^ and 1023 cm^−1^ are related with the stretching vibration of C-O-C bound. The spectrum of chitosan with TiO_2_ nanoparticles (grey curve, Figure 3) exhibits bands related to Ti-O bonding at approximately 635 cm^−1^ and 1386 cm ^−1^ [33,34]. It should be noted that the addition of TiO_2_ nanoparticles causes an increase in intensity of the signal in the range of 3500–2750 cm^−1^, corresponding to the O-H, N-H and C-H groups. In addition to this, a strong enhancement and broadening of the bands for N-H and C-N is visible. Compared with the spectrum of the chitosan coating, the broader and stronger peaks are shifted to lower wavenumbers for the chitosan/TiO_2_ coating (for example, from 3358 cm^− 1^ to 3183 cm^−1^, from 2872 cm^−1^ to 2860, and from 1596 cm^−1^ to 1542 cm^−1^, for chitosan and chitosan/TiO_2_ coatings, respectively), indicating that there is an interaction between the groups O-H, N-H, C-N, and the TiO_2_ nanoparticles. These results showed that -OH and/or -NH_2_ groups from the chitosan chains may interact with the TiO_2_ nanoparticles to form covalent bonds or hydrogen bonds [35]. Such an interaction of chitosan molecules with TiO_2_ nanoparticles was observed in the reference [36]. The characteristic bands observed in the range from 700 to 500 cm^−1^ are attributed to the Ti-O-C bending mode and Ti-O-Ti stretching vibration. The interaction of TiO_2_ nanoparticles with functional groups present in the chitosan chain ensures their uniform distribution in the polymer matrix (chitosan matrix).

**Figure 4:**
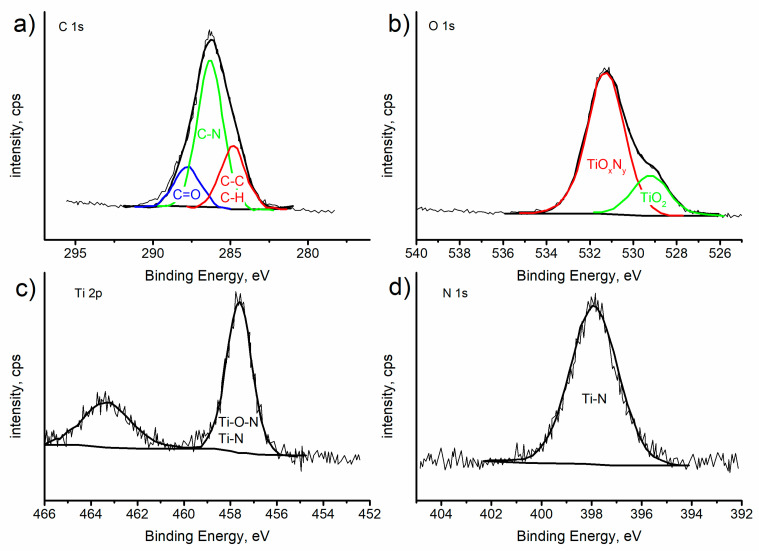
XPS spectra recorded for chitosan/TiO_2_ coatings deposited on the surface of Mg19Zn1Ca alloy. XPS deconvoluted profiles for C1s (**a**), O1s (**b**), Ti2p (**c**), and N1s (**d**).

SEM observations (Figure 2e,g) and the features observed in the infrared spectra support the existence of TiO_2_ nanoparticles–chitosan matrix interactions. Such results were confirmed by X-ray photoelectron spectroscopy (XPS) measurements performed on the chitosan/TiO_2_ coating deposited on the substrate. The chitosan/TiO_2_ coating consists of 56.3 at.% carbon, 34.8 at.% oxygen, 7.2 at.% nitrogen, and 1.7 at.% Ti. Figure 4 presents the XPS spectra recorded for the chitosan/TiO_2_ coating in the individual bands C1s, O1s, N1s, and Ti2p. The peaks present in the C1s, O1s and N1s photoelectron spectra are related with the chemical bounds present in the chitosan molecule [37]. Multiple peaks fitted from the C1s spectrum (Figure 4a), the binding energy of 284.8 eV, 286.3 eV, 287.8 eV correspond to C-C/C-H, C-N and C=O chemical bounds, respectively. In the O1s spectrum (Figure 4b), the two peaks at the binding energy of 529.2 eV and 531.3 eV can be assigned to oxide ions in TiO_2_ and titanium oxynitride TiO_x_N_y_, respectively [38]. The single peak fitted from Ti2p XPS spectrum located at the binding energy 457.6 eV is associated with the presence of Ti (III) in the titanium oxynitride Ti-O-N or Ti-N, Figure 4c [39,40,41]. The peak located at 398.0 eV recorded in the N1s spectrum (Figure 4d) is assigned to Ti-N bound [39,40].

### 2.2. Corrosion Resistance of Uncoated and Coated Mg19Zn1Ca Alloys

**Figure 5:**
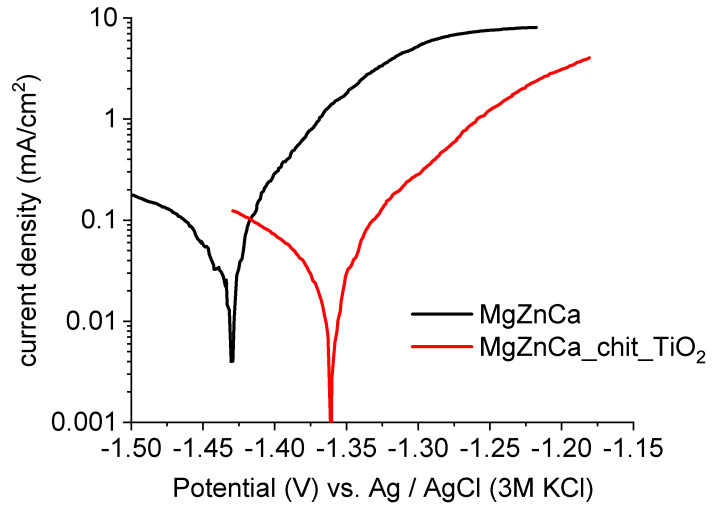
Linear sweep voltammetry (LSV) curves (1 mV/s) performed for uncoated (black curve) and coated Mg19Zn1Ca alloys (red curve) in Hank’s solution.

Figure 5 shows the polarisation curves of both the Mg19Zn1Ca alloy (substrate) and the substrate covered by the chitosan/TiO_2_ coating. Both polarisation curves have a similar shape. A sharp increase in the current density is observed with increasing applied potential in the anodic domain (active behaviour). However, the current density values obtained for the specimen covered by the chitosan/TiO_2_ coating (red curve in Figure 5) are significantly lower than those obtained for the MgZnCa substrate. For example, the anodic current density registered at the potential of −1.3 V (the potential located in the anodic domain) is 5.2 mA/cm^2^ for the MgZnCa alloy, and only 0.28 mA/cm^2^ for the magnesium alloy covered by the chitosan/TiO_2_ coating. The corrosion potential of the magnesium alloy coated with the chitosan/TiO_2_ coating is shifted toward higher values (of about 0.07 V) compared to that recorded for the pure substrate. Therefore, the presence of the chitosan/TiO_2_ coating has a beneficial influence on the kinetics of anodic reactions. These results show that the specimen covered by the chitosan/TiO_2_ coating has the highest corrosion resistance in Hank’s solution.

These results were confirmed by determining the numerical values of the polarisation resistance (R_p_) using the linear polarisation resistance (LPR) method [42]. LPR measurements were performed in Hank’s solution. The potential scan rate was set at 0.125 mV/s and the potential range was [−25, +25] mV vs. OCP (OCP was previously measured for 30 min). R_p_ was found to be equal to 214 ± 60 Ω.cm^2^ and 952 ± 250 Ω.cm^2^ for the uncoated and coated magnesium alloys, respectively. Therefore, the value of R_p_ is increased by a factor of 4.4 in the presence of the chitosan/TiO_2_ coating. These results confirm those derived from the polarisation curves, indicating that the Mg19Zn1Ca alloy covered by chitosan/TiO_2_ coating exhibits significantly better corrosion resistance in the Hank’s solution compared to the uncoated specimen.

### 2.3. The Influence of the Chitosan/TiO_2_ Coating on Corrosion Mechanisms

**Figure 6:**
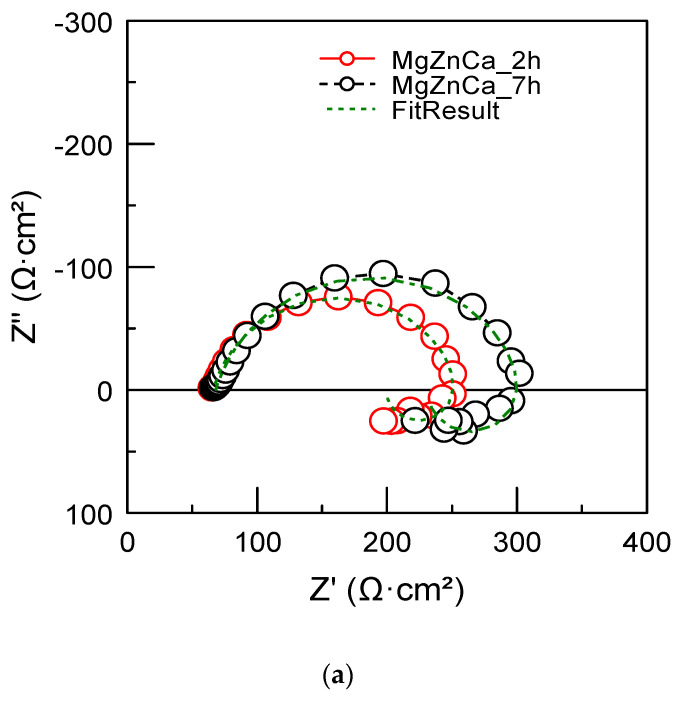

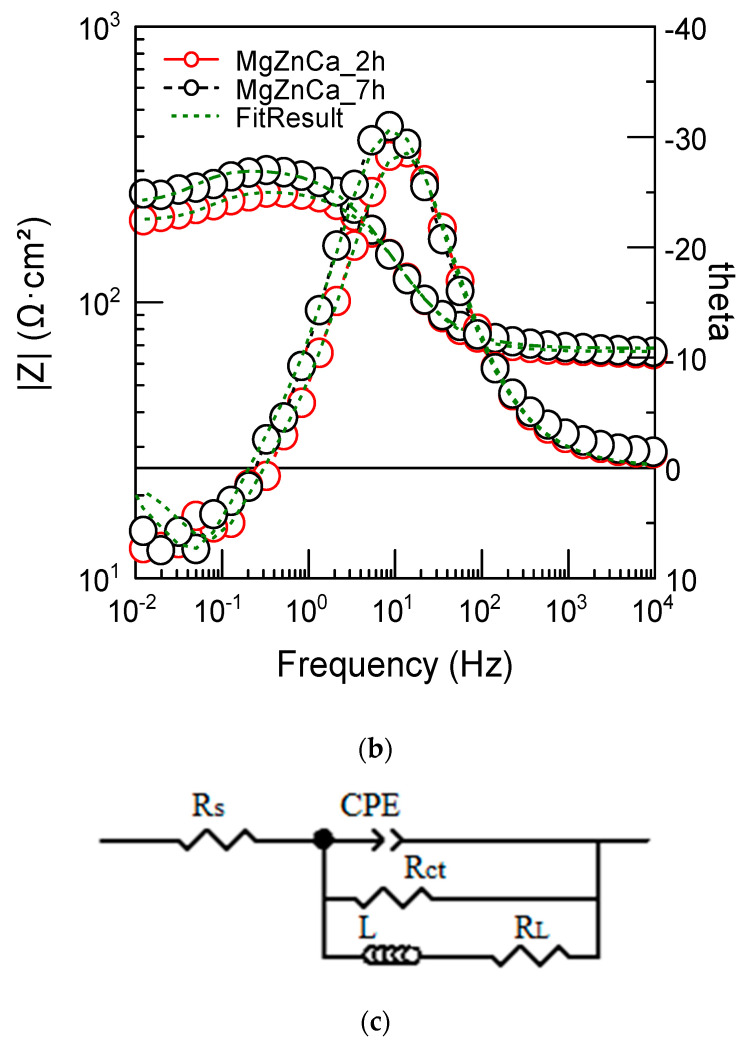
The EIS spectra measured after immersion of the MgZnCa alloy for 2 and 7 h in Hank’s solution, (**a**) Nyquist plots, (**b**) Bode diagram, and (**c**) electrical equivalent circuit used for fitting the experimental spectra.

Electrochemical impedance spectroscopy (EIS) is considered as a non-destructive method to investigate the corrosion mechanisms of alloys [42]. Figure 6a shows the EIS spectra for the MgZnCa alloy immersed in the Hank’s solution at the OCP value for 2 and 7 h. These spectra were fitted using the electrical equivalent circuit shown in Figure 6b. It is composed of the solution resistance (Rs) at high frequency and a combination of components (CPE//R_ct_//L- R_L_) at medium and low frequencies. CPE is the constant phase element (corresponding to the formation of oxides at the interface solution/alloy) R_ct_ is the charge transfer resistance, L is an inductance, and R_L_ is an inductance resistance. The constant phase element (CPE) is used to simulate the capacitive response of non-ideal capacitor, due to the heterogeneous nature of the electrode surface. The impedance of the constant phase element (CPE) is given in Equation (1).

(1) $$Z_{CPE}=\frac{1}{{Y\left(j\omega \right)}^{n}}$$

where Y is the value of admittance expressed in (S cm^−2^ s^n^), ω is the angular frequency (rad s^−1^), j is the imaginary number (j^2^ = (−1)) and n is a dimensionless fractional exponent. When n = 1, 0, or −1, the CPE represents an ideal capacitor, a resistance and an inductor, respectively. The inductive loop in the low-frequency range is mainly associated with the adsorption of corrosion products and/or pitting corrosion initiation [43,44,45,46]. The numerical values of the components of the equivalent circuit are listed in Table 1.

A slight increase in the charge transfer resistance (R_ct_) and the inductance resistance (R_L_) is observed with increasing immersion time (by a factor of ~1.2). The incubation time T_L_ of pitting corrosion was calculated from the numerical values of the components of the inductive loop [47], Equation (2).

(2) $$T_{L}=\frac{L}{R_{L}}$$

T_L_ was also found to slightly increase with increasing immersion time (from 2.6 s after 2 h immersion up to 4.6 s after 7 h immersion). Therefore, the adsorption of corrosion products on the specimen surface partly inhibits corrosion mechanisms and slightly retards the initiation of pitting corrosion. Corrosion products are mainly composed of Mg_3_(PO_4_)_2_ and CaCO_3_, MgCO_3_ [5,6].

**Figure 7:**
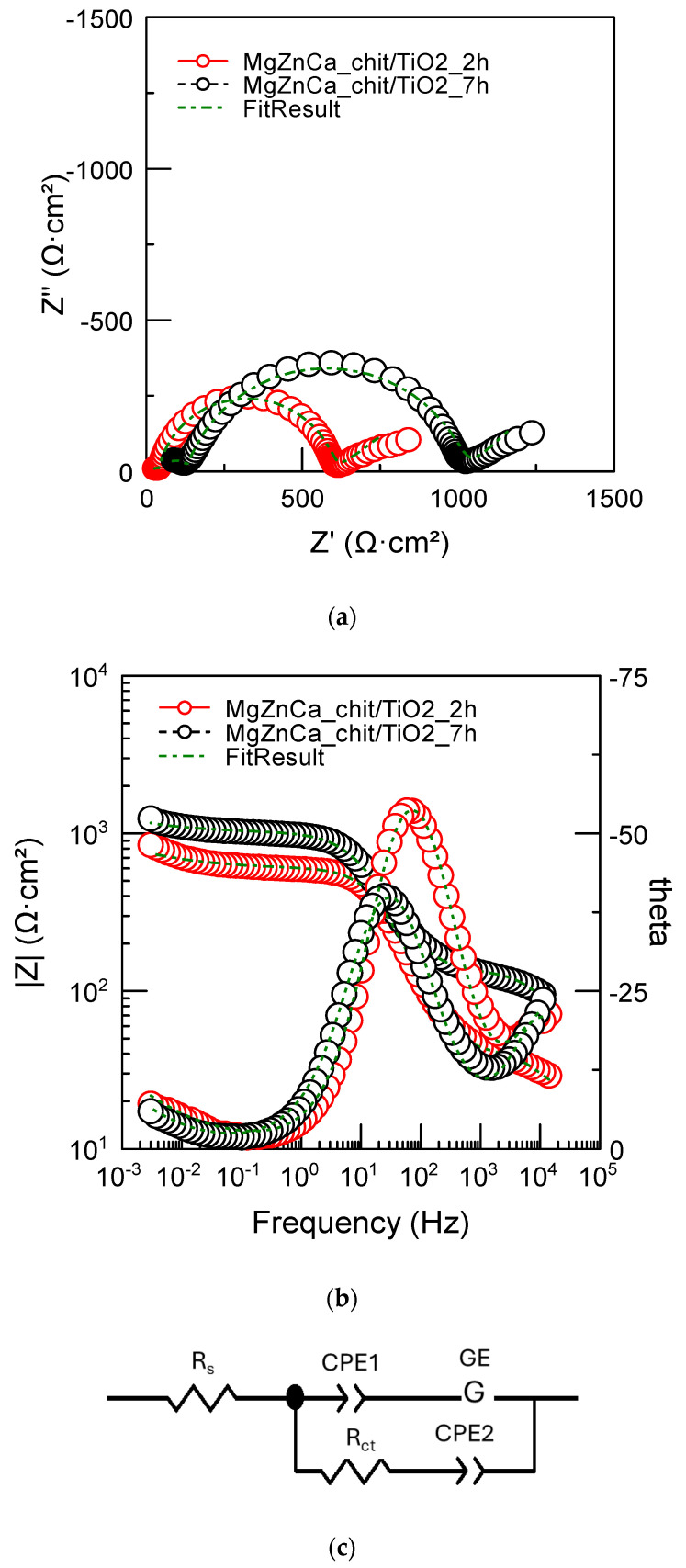
EIS spectra performed for Mg19Zn1Ca_chit/TiO_2_ coated alloy after immersion for 2 and 7 h in Hank’s solution. (**a**) Nyquist plots, (**b**) Bode plot, and (**c**) electrical equivalent circuit used for fitting the experimental spectra.

Figure 7a shows EIS spectra obtained for the Mg19Zn1Ca alloy coated by the chitosan/TiO_2_ coating (after immersion for 2 and 7 h). In the presence of the coating, EIS spectra are different from those obtained on the uncovered alloy. They were fitted considering the equivalent circuit shown in Figure 7b. R_s_ is again the solution resistance and R_ct_ the charge transfer resistance. The exponent of CPE1 is close to unity (value of 0.9 in Table 2), indicating that it can be considered as a capacitor (capacitive loop observed at medium frequencies). By contrast, the exponent of CPE2 is close to 0.5, indicating that it corresponds to a diffusion (low-frequency range). The corrosion processes were then controlled by the diffusion of species through the chitosan/TiO_2_ coating [48]. In contrast to the previous case (uncoated substrate), the value of R_ct_ increases significantly (by a factor of 1.6) with increasing immersion time. Such increases in R_ct_ are related to the deposition of corrosion products on the surface of the coated specimen. During coating degradation, the alloy is dissolved, and corrosion products are deposited on the electrode surface. The Gerischer impedance was used for describing the diffusion of species involved in the chemical–electrochemical reactions’ mechanisms from the bulk of the solution to the electrode–solution interface. The Gerischer element is given by G = Y(k + jω) ^0.5^, where k is the rate constant parameter. Magnesium cations diffuse through the chitosan/TiO_2_ coating, then react with phosphate ions present in Hank’s solution. In Hank’s solution, calcium ions react with the carbonate’s ions and then the CaCO_3_ is deposited on the electrode surface. Magnesium phosphates and carbonates are deposited on the surface of the coated alloy and hinder further dissolution of the magnesium alloy.

### 2.4. Corrosion Rate of Uncoated and Coated Mg19Zn1Ca Alloy

**Figure 8:**
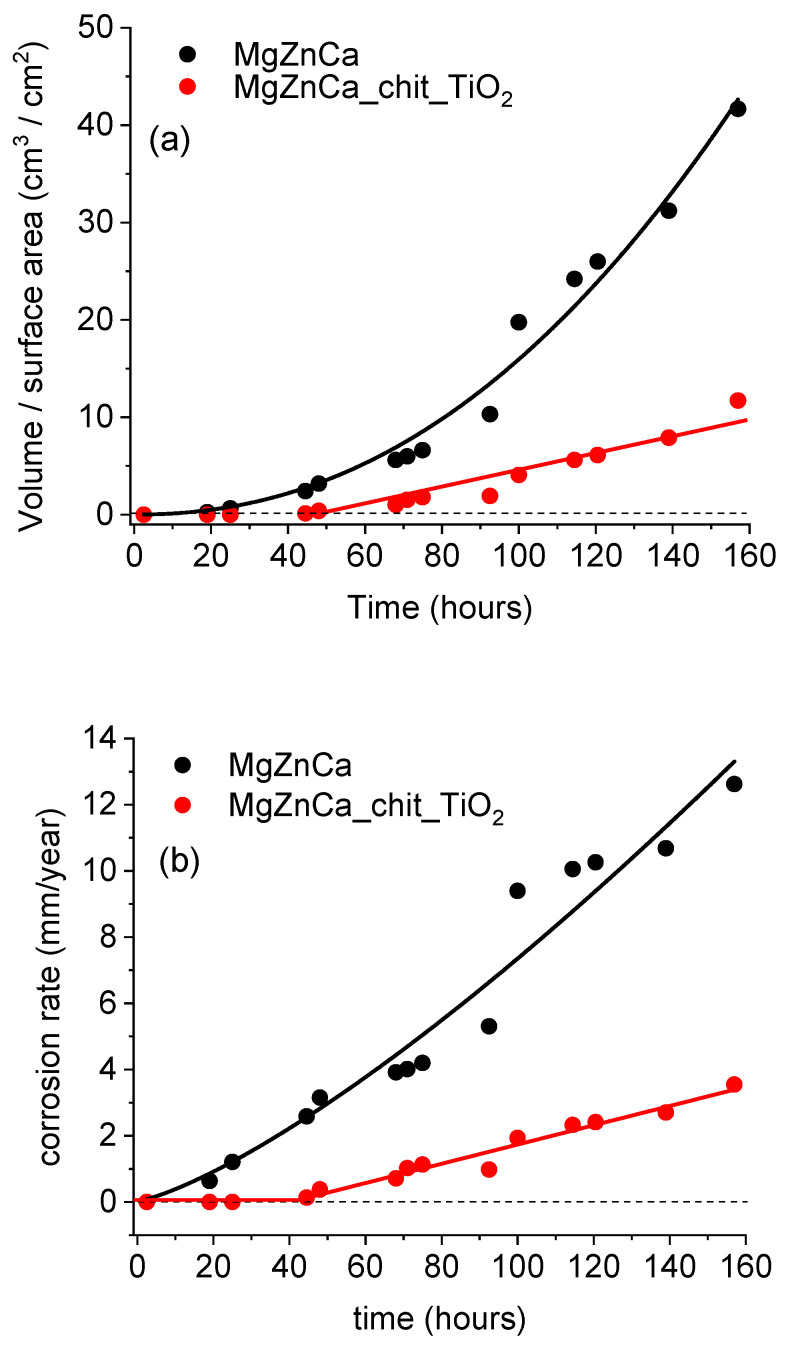
Evolution vs. time of (**a**) the ratio between the volume of released hydrogen and sample surface area and (**b**) the corrosion rate for the uncoated and coated specimens soaked at the OCP in Hank’s solution (**a**).

The corrosion rate of the uncoated and coated magnesium alloys was calculated by collecting the hydrogen evolved during the corrosion process. This was performed by applying the method described in reference [49,50]. The samples were immersed at the OCP for 160 h in Hank’s solution at 37 °C. Figure 8a shows the evolution vs. time of the ratio τ between the volume of released hydrogen and the specimen surface area for the two specimens.

For the uncoated sample, hydrogen is produced after short-term immersion. This indicates that corrosion of the uncoated substrate starts immediately after its immersion in the Hank’s solution. The overall corrosion reaction of magnesium in aqueous solutions is expressed by reaction (3):

(3) $$Mg+{2H}_{2}O\to {Mg\left(OH\right)}_{2}+H_{2}$$

The ratio τ was found to increase sharply with time, according to a power function (black curve in Figure 8a). By contrast, for the substrate covered by the chitosan/TiO_2_ coating, the volume of released hydrogen was measurable only after 45 h of immersion. This shows that the coating protects the substrate against corrosion after short-term immersion. From 45 h of immersion, the ratio τ increases slowly and linearly vs. time (red curve in Figure 8a). It can be observed that the value of τ after 160 h of immersion is four times greater in the absence of coating. This result shows clearly that the presence of the chitosan/TiO_2_ coating has a beneficial influence on the corrosion resistance of the magnesium alloy.

Equation (4) was used to calculate the corrosion rate of uncoated and chitosan/TiO_2_-coated magnesium alloy [51].

(4) $$v=87.6\left(\frac{m\left[mg\right]}{d\left[\frac{g}{{cm}^{3}}\right]\cdot s\left[{cm}^{2}\right]\cdot t\left[h\right]}\right)\left[mm/y\right]$$

where d is the density of magnesium, s is the surface area of metal exposed to the corrosive environment, t is the time, and m is the mass of magnesium dissolved during the corrosion test. Considering reaction (3), the dissolution of 1 mole of magnesium causes the release of 1 mole of hydrogen. Thus, knowing the volume of hydrogen released during the corrosion test, the mass of the dissolved magnesium can be calculated. Figure 8b shows the evolution of the corrosion rate vs. time for the two samples. As was already observed from Figure 8a, corrosion proceeds immediately on the uncovered sample, whereas it starts on the covered sample after 45 h of immersion. For long immersion times (greater than 45 h), the corrosion rate of the covered sample increases slowly vs. time, whereas that of the uncovered sample increases sharply. After 157 h of immersion of the two specimens in Hank’s solution, the corrosion rate was 12.6 and 3.5 mm/y for uncoated and coated substrates, respectively.

**Figure 9:**
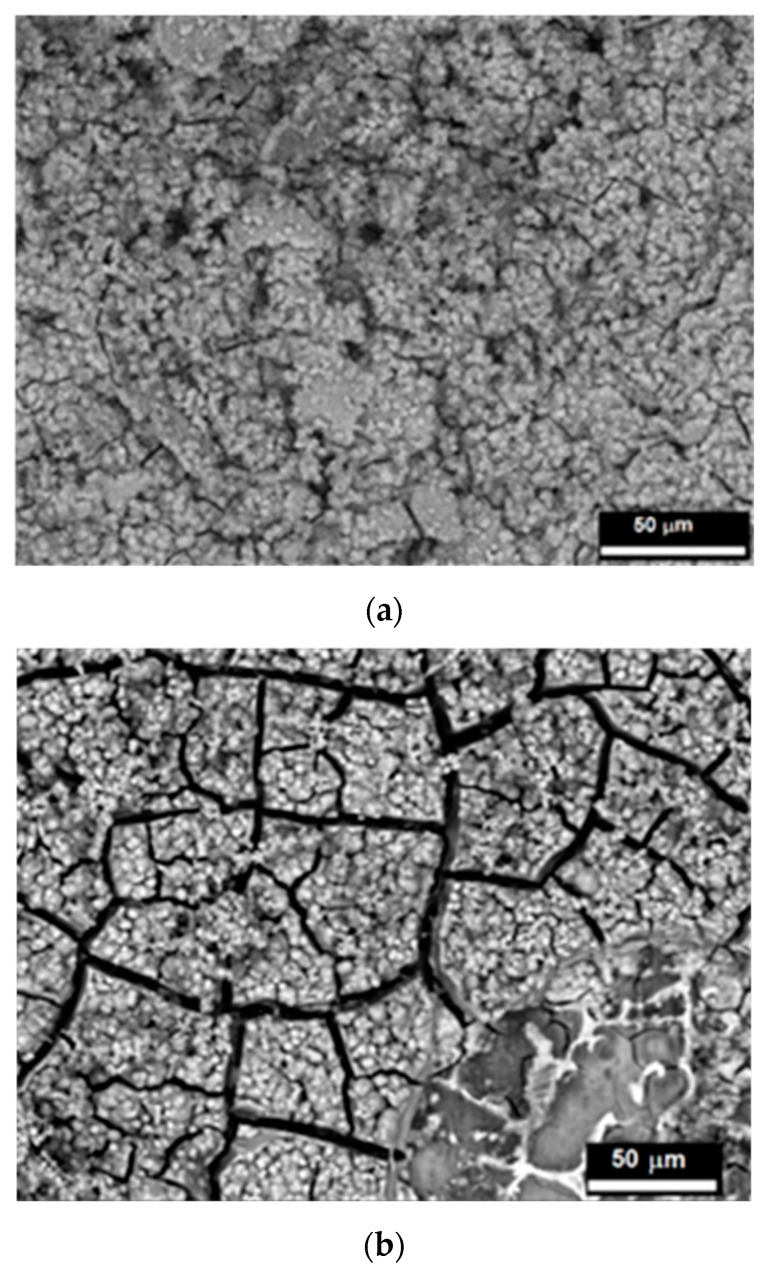
SEM image of the coated Mg19Zn1Ca alloy after immersion for 24 h (**a**) and 4 days (**b**) in Hank’s solution.

Figure 9 depicts the surface morphology of the chitosan/TiO_2_-coated Mg19Zn1Ca alloy, immersed in Hank’s solution for 24 h and 4 days, respectively. After 24 h of immersion in Hank’s solution, some small micro-cracks are visible on the surface of the chitosan/TiO_2_-coated Mg alloy (Figure 9a). However, after a longer immersion time, numerous and wide micro-cracks appear on the surface of the Mg19Zn1Ca alloy covered with the chitosan/TiO_2_ coating (Figure 9b). Wide micro-cracks are caused by the presence of internal stresses in the layer of corrosion products that grows over time.

EDS analysis performed after exposition of the specimen in Hank’s solution for 24 h revealed the presence of the following elements: carbon (7.2 at.%), nitrogen (13 at.%), oxygen (55 at.%), magnesium (12.5 at.%), phosphorous (5.0 at.%), chloride (1.5 at.%), calcium (1.3 at.%), titanium (2.5 at.%), and traces of zinc (below 1 at.%). The presence of nitrogen, carbon and titanium confirms that the chitosan/TiO_2_ coating was not destroyed. It is still present on the substrate surface. As shown in Figure 9, after 24 h of immersion in Hank’s solution by the coated alloy, there is no hydrogen evolution, and the corrosion rate is equal to 0 mm/year.

SEM/EDS analyses performed on the coated specimen exposed for 4 days in the corrosive environment reveal the presence of carbon (7.2 at.%), oxygen (65.3 at.%), magnesium (4.3 at.%), phosphorus (10.0 at.%) and calcium (12.0 at.%). The absence of nitrogen and titanium indicates that the coating was partly destroyed. The large amount of phosphorus and calcium compared to those found on the sample soaked for 24 h indicates that a layer of corrosion products was formed on the sample surface. SEM observations show the presence of cracks in the corrosion products caused by the presence of high levels of internal stress (Figure 9b).

**Figure 10:**
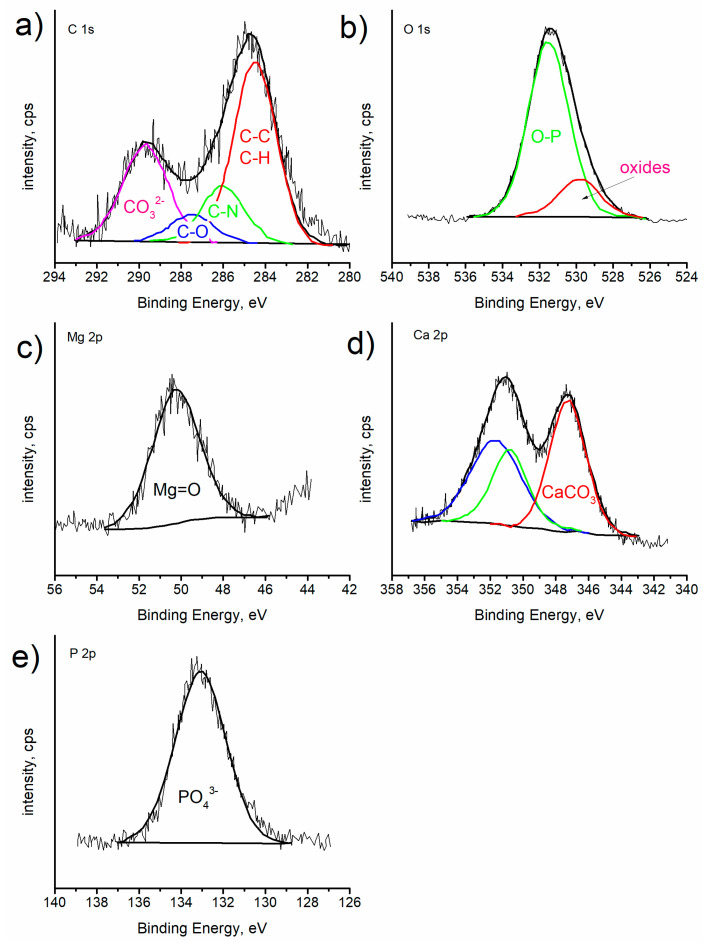
XPS spectra recorded for chitosan/TiO_2_ coatings deposited on the surface of Mg19Zn1Ca alloy. XPS deconvoluted profiles for C1s (**a**), O1s (**b**), Mg2p (**c**), Ca2p (**d**), and P2p (**e**).

The chemical composition of corrosion products formed after the 4-day immersion of the coated sample was studied by means of XPS. After the corrosion test, the following elements were detected: 20.7 at.% carbon, 53.8 at.% oxygen, 8.0 at.% magnesium, 9.3 at.% calcium, and 7.0 at.% phosphorus. Figure 10 shows the XPS spectra recorded in the individual bands C1s, O1s, Mg2p, Ca2p and P2p. The peak related to the C1s level was fitted considering four components as shown in Figure 10a. The components located at 284.5 eV, 286.0 eV, 287.5 eV, and 289.7 eV are related to C-C/C-H, C-N, C=O and carbonates, respectively [52,53,54]. For the O1s spectrum (Figure 10b), two main components were observed at 529.8 eV and 531.5 eV, which are assigned to oxides and O-P bound, respectively [55]. The peak associated with the Mg 2p level at the binding energy of 50.25 eV is related with MgO [56]. The P 2p spectrum was fitted with one peak located at the binding energy of 133.0 eV (phosphate species). The Ca 2p spectrum shows one doublet (Ca2p1/2 at 350.8 eV and Ca2p3/2 at 347.3 eV) (Figure 10d). The peak located at the binding energy of 347.3 eV is attributed to calcium carbonate (CaCO_3_) [55]. Additionally, the peak at the binding energy 289.7 eV located in the C1s spectrum confirms the presence of carbonates in the layer of corrosion products. XPS analysis revealed that the main corrosion products deposited on the surface of the coated magnesium alloy are calcium carbonates and magnesium phosphates.

### 2.5. Corrosion Degradation of Uncoated and Coated Mg19Zn1Ca Alloy

The corrosion of magnesium alloy in Hank’s solution proceeds according to the reactions (5)–(7).

(5) $$Mg\to {Mg}^{2+}+2e$$

(6) $$2H_{2}O+2e\to H_{2}+{2OH}^{-}$$

(7) $${Mg}^{2+}+{2OH}^{-}={Mg\left(OH\right)}_{2}$$

The anodic dissolution of magnesium causes the formation of Mg^2+^ ions, reaction (5). The electrons released during the anodic reaction then participate in the reduction in the water molecule—reaction (6). During the cathodic reaction, the hydrogen gas is evolved and OH- ions are formed. Then, magnesium cations Mg^2+^ react with the OH^−^ anions and the main corrosion product Mg(OH)_2_ is formed—reaction (7). The overall corrosion reaction of magnesium and its alloys is given by Equation (3). Moreover, the phosphate ions present in the Hanks’ solution can react with the magnesium ions—reaction (8). Calcium ions react with carbonate ions in the solution and form insoluble salt according to reaction (9).

(8) $${3Mg}^{2+}+{2PO}_{4}^{3-}\to {Mg}_{3}{\left({PO}_{4}\right)}_{2}$$

(9) $${Ca}^{2+}+{CO}_{3}^{2-}\to {CaCO}_{3}$$

Magnesium phosphate and calcium carbonate undergo deposition on the surface of the magnesium alloy and form the barrier layer. Such a barrier layer (corrosion products) hinders further dissolution of the magnesium alloy. As shown in Figure 8b (black curve), the corrosion rate of uncoated Mg19Zn1Ca alloy varies with exposure time. For immersion times up to 93 h, the corrosion rate increases linearly with time and the slope of the curve is 0.061; for immersion times longer than 100 h, the slope of the black curve is 0.052. The decrease in the slope value suggests that corrosion products are deposited on the surface of the magnesium alloy and slow down its dissolution.

**Figure 11:**
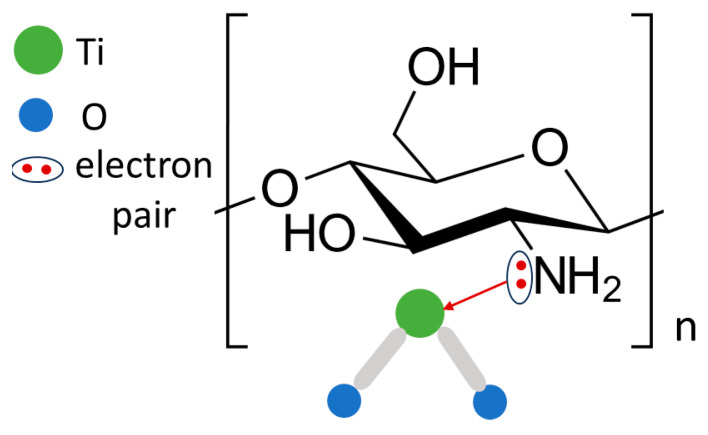
The interaction of TiO_2_ (NPs) with the chitosan molecule.

In the case of the coated specimen, the corrosion rate of the substrate significantly decreased. This was related with the interaction of TiO_2_ (NPs) with chitosan molecules and forming a protective barrier layer. XPS analysis has confirmed the presence of Ti-N and Ti-O-N bounds in the coating. In the FTIR spectrum, the characteristic band attributed to Ti-O-C bending mode was detected; the interaction of the TiO_2_ (NPs) caused their uniform distribution in the chitosan matrix. Therefore, the specific chitosan/TiO_2_ network was formed (Figure 11). The incorporation of TiO_2_ (NPs) in the chitosan matrix is related to complexation by amino NH_2_ groups. The lone pair of electrons from nitrogen is shifted to the titanium atom and a stable coordinate structure is formed. Such a structure reduced the surface of substrate exposed to Hank’s solution and slowed down its corrosion degradation.

However, after 4 days of immersion of the coated specimen in Hank’s solution, the chitosan/TiO_2_ coating was partly destroyed, and the corrosion products were formed at the bare alloy. XPS analysis showed that there was a significant decrease in carbon (20.7 at.%) after 4 days of exposure in Hank’s solution of the coated alloy compared to the carbon content (56.3 at.%) in the chitosan/TiO_2_ coating before the corrosion test. Furthermore, the lack of signal in the N1s and Ti2p bands indicates that the chitosan/TiO_2_ coating was incompletely, but significantly, damaged. The exposed parts of the alloy begin to corrode, and deposition of corrosion products occurs. The formation of the corrosion products proceeds according to the reactions (8)–(9). The formation of corrosion products such as calcium carbonates and magnesium phosphates has been confirmed by XPS analysis (Section 3.4).

The corrosion rate of the coated sample varies linearly with time, as shown in Figure 8b. Unlike the bare alloy (black curve), the slope of the red curve (coated alloy) remains constant over the time range from 40 to 157 h. The slope calculated for the red curve is 0.027, (Figure 8b). The value of the slope of the red curve obtained for the coated alloy being two times lower than the uncoated alloy indicates that the chitosan/TiO_2_ coating limits the contact of the substrate with Hank’s solution and significantly reduces its corrosion.

## 3. Materials and Methods

### 3.1. Materials

Mg19Zn1Ca (in wt%) alloy was delivered by Goodfellow in the form of rods. The samples were prepared for corrosion tests in the form of cylinders with a diameter of 10 mm and a height of 10 mm. The electrical contact was made using an electric cable that was soldered to the lateral surface of the alloy, and the samples were then embedded in an epoxy resin. The flat surface of the alloy immersed in the electrolyte solution was 0.785 cm^2^.

### 3.2. Coatings Preparation

Prior to the deposition of coatings, specimens were ground with sandpaper (1200 grit), cleaned by sonication for 300 s in ethanol, and then dried with argon. The chitosan solution containing titanium dioxide (TiO_2_) nanoparticles was prepared in the following procedure. Two solutions were first prepared. In solution (1), 2 g of chitosan powder was dissolved in 1 vol.% of acetic acid. This solution was stirred at 250 rpm for 24 h. In solution (2), 0.5 g of TiO_2_ nanoparticles (average size 20 nm) was dissolved in 50 mL of 1% vol. acetic acid. Then, 50 mL of both solutions were mixed. This mixed solution (chitosan/TiO_2_) was used for deposition of coatings on the Mg19Zn1Ca alloy substrate. The chitosan/TiO_2_ coatings were deposited using the spin coater POLOS SPIN 150i. The procedure used for the deposition of chitosan/TiO_2_ coatings consists of four steps, the details of deposition conditions are given in Table 3. Six layers of chitosan/TiO_2_ coating were deposited to the surface of the Mg19Zn1Ca alloy (the procedure of deposition is presented in Table 3 was repeated 6 times).

The chitosan powder used for the preparation of chitosan/TiO_2_ coatings was delivered by Acros Organics. Its molecular mass was 100–300 kDa and degree of deacetylation 75–85%. TiO_2_ nanoparticles (NPs) were delivered by Sigma–Aldrich. The average size of TiO_2_ (NPs) was 20 nm.

### 3.3. Corrosion Measurements

Prior to the corrosion tests, the surface preparation described in Section 2.2 was applied to the uncoated samples. The corrosion tests were carried out on uncoated and coated Mg19Zn1Ca substrate in Hank’s solution. The chemical composition of the Hank’s solution is: 8 g/L NaCl, 0.4 g/L 1.41 KCl, 0.140 g/L CaCl_2_, 0.350 g/L NaHCO_3_, 0.217 g/L NaH_2_PO_4_, 0.06 g/L Na_2_HPO_4_, 0.406 g/L 1.42 MgCl_2_•6H_2_O, 0.029 g/L MgSO_4_, and 1 g/L D-glucose. A classical electrochemical three-electrode cell containing (Ag/AgCl (3M KCl)) a reference electrode, a Pt plate (surface area of electrode 4 cm^2^) as a counter-electrode, and the specimen as a working electrode was used for performing the corrosion measurements. PGSTAT128 Potentiostat/Galvanostat with Nova 2.1 software (Metrohm Autolab, Utrecht, The Netherlands) was used to perform the corrosion tests.

Prior to electrochemical measurements, such as linear sweep voltammetry (LSV) and linear polarization resistance (LPR), the open circuit potential (OCP) was measured for 30 min (to reach steady state). LSV curves were plotted from −50 mV vs. OCP to the anodic direction. A potential scan rate of 1 mV/s was used to plot the polarization (LSV) curves. For LPR measurements, the potential was scanned at a rate of 0.125 mV/s, in the potential range from an initial value of -20 mV vs. OCP to a final value of +20 mV vs. OCP. These measurements (LSV and LPR) were performed for uncoated and coated Mg19Zn1Ca alloy. All the corrosion tests were performed in Hank’s solution (T = 37 °C, pH = 7.2).

Electrochemical Impedance Spectroscopy (EIS) measurements were performed at the open circuit potential (OCP) in Hank’s solution. A sinusoidal AC wave of ±10 mV amplitude was applied from 10 kHz to 0.008 Hz. Fitting of the experimental spectra was carried out using the ZView 4 software (Scribner Associates, Southern Pines, NC, USA).

The method described in [49] was used to calculate the corrosion rate of bare magnesium alloy and magnesium alloy coated with chitosan/TiO_2_. The samples were immersed in Hank’s solution (T = 37 °C, pH = 7.2) for 6 days. During corrosion of the magnesium alloy, hydrogen was released, which was collected in a burette placed on the surface of the test sample. A detailed calculation of the corrosion rate based on the amount of hydrogen released during the corrosion test is described in paragraph 3.4.

### 3.4. Characterisation Techniques

Prior to microstructural characterisations, uncoated samples were mechanically ground using emery papers (800 down to 4000) and then polished with the SiO_2_ suspension. Top-view and cross-section surface imaging of the uncoated and coated specimens were carried out with FE-SEM/EDS (JEOL, JSM-5500LV, Tokyo, Japan). The phase composition of the Mg19Zn1Ca alloy was investigated by X-ray diffraction (XRD, Philips PW-3710 X’PERT diffractometer, Philips Analyical, Almelo, The Netherlands), using Cu-Kα radiation with a step size of 0.026° in the 2θ range of 10–100°. FTIR spectra of the chitosan-based coatings were obtained by using the Thermo-Scientific Nicolet iS50 FT-IR spectrophotometer (Thermo Fisher Scientific, Waltham, MA, USA). This spectrometer was equipped with an attenuated total reflectance accessory (ATR). FTIR spectra were measured over a scan range of 4000 to 500 cm^−1^, and 124 scans were performed. The roughness of the coating was measured by using Dektak 150 Surface Profiler (Veeco Instruments Inc., Plainview, NY, USA).

The chemical states of the elements present on the surface of chitosan/TiO_2_ coatings were characterised by X-ray photoelectron spectroscopy (XPS apparatus, SIA 130 100 [Cameca Riber, Madison, WI, USA], and X-ray beam diameter of 200 μm). XPS spectra were obtained with the following parameters: nonmonochromatic Al Ka line, energy of 1486.7 eV, power of 50 W, 15 kV voltage, and pass energy 187 eV/58.7 eV for spectra/windows. A Mac 2 semi-imaging spectrometer was used with a resolution of 1.3 eV (width of Ag 3d5/2 level). The carbon peak at the binding energy at 285 eV was considered for energy calibration. XPS analysis was performed after the chitosan/TiO_2_ coating was deposited on the surface of the Mg19Zn1Ca alloy, and after corrosion testing of the coated alloy. The coated magnesium alloy was soaked in Hank’s solution for 4 days, then the sample was removed from the corrosion environment, air-dried for 24 h, and XPS analysis was performed. XPS spectra were analysed using the CasaXPS version 2.3.25 software package.

## 4. Conclusions

The chitosan/TiO_2_ coating was deposited on the Mg19Zn1Ca alloy to protect it against corrosion in Hank’s solution. Magnesium alloy (Mg19Zn1Ca) undergoes active corrosion in Hank’s solution. The corrosion products, such as Mg(OH)_2_ and Mg_3_(PO_4_)_2_, are deposited on the surface of the magnesium alloy, forming a barrier layer. This layer slowed down further dissolution of the alloy. The chitosan/TiO_2_ coatings deposited on the surface of Mg19Zn1Ca alloy significantly enhance its corrosion resistance in the Hank’s solution. In the Hank’s solution, the corrosion rate of the coated alloy is 3.6 times lower than the corrosion rate of the uncoated alloy. The interaction of the TiO_2_ nanoparticles (NPs) with the amine groups present in the chitosan molecule caused their uniform distribution in the chitosan matrix. Therefore, the specific chitosan/TiO_2_ network was formed, which reduced the surface of substrate exposed to Hank’s solution and slowed down its corrosion degradation. Degradation of the chitosan/TiO_2_ coating is controlled by the diffusion of magnesium ions through the coating and deposition of corrosion products.

## Figures and Tables

**Table 1:** Fitting results obtained from the EIS spectra of MgZnCa alloy with immersion time in the Hank’s solution.

	R_s_ (Ω·cm^2^)	R_ct_ (Ω·cm^2^)	Y (S·cm^−2^·s^n^)	n	R_L_ (Ω·cm^2^)	L (H·cm^2^)
2 h	66.0 ± 0.4	191.9 ± 1.8	2.4 × 10^−4^ ± 4 × 10^−6^	0.84 ± 0.009	436.5 ± 21	1117.0 ± 87
7 h	68.0 ± 0.67	239.6 ± 2.5	2.9 × 10^−4^ ± 1.3 × 10^−5^	0.84 ± 0.003	489.5 ± 27.0	2229.0 ± 127.0

**Table 2:** Fitting results obtained from the EIS spectra of the MgZnCa alloy coated by chitosan/TiO_2_ coating with immersion time in Hank’s solution.

	R_s_ (Ω·cm^2^)	R_ct_ (Ω·cm^2^)	Y1 (S·cm^−2^·s^n^)	n1	Y2 (S·cm^−2^·s^n^)	n2	GE-T (S·cm^−2^·s^0.5^)	GE-P
2 h	13.2 ± 2.0	585.9 ± 4.9	2.6 × 10^−5^ ± 2.2 × 10^−7^	0.9 ± 0.005	0.029 ± 4.4 × 10^−4^	0.42 ± 0.017	2.1 × 10^−4^ ± 1.2 × 10^−5^	2938 ± 946
7 h	33 ± 0.8	991.2 ± 7.3	3.4 × 10^−5^ ± 3.8 × 10^−7^	0.82 ± 0.006	5.8 × 10^−5^ ± 2.2 × 10^−7^	0.49 ± 0.02	5.8 × 10^−5^ ± 3.0 × 10^−6^	27,652 ± 449

**Table 3:** Procedure used for the deposition of chitosan/TiO_2_ coatings by using spin coated method.

Step	Time, s	Speed, rps
S1	5	250
S3	30	1000
S3	60	1500
drying	60	250

## Data Availability

Any data or material that support the findings of this study can be made available by the corresponding author upon request.
